# Transarterial embolization for an infected internal iliac artery aneurysm without recurrence for a long period: A case report

**DOI:** 10.1016/j.radcr.2024.04.035

**Published:** 2024-05-11

**Authors:** Atsuhiro Ijiri, Yohsuke Suyama, Osamu Ishida, Koji Sumi, Koji Tsutsumi, Hiroshi Shinmoto

**Affiliations:** a Department of Radiology, National Defense Medical College, 3-2 Namiki, Tokorozawa, Saitama 359-8513, Japan; b Department of Cardiovascular Surgery, National Defense Medical College, 3-2 Namiki, Tokorozawa, Saitama 359-8513, Japan

**Keywords:** Infected aneurysm, Iliac artery aneurysm, Endovascular treatment, Coil embolization, Open surgery, Transarterial embolization

## Abstract

We report an 85-year-old man who underwent transarterial embolization (TAE) for an infected internal iliac artery aneurysm. The patient presented with fever and left lower abdominal pain. Computed tomography (CT) revealed the expansion of a left internal iliac artery aneurysm. We planned surgical treatment for an infected internal iliac artery aneurysm; however, the patient's age and general condition made the surgery high-risk. Therefore, we performed emergency TAE. The patient was administered antibiotics for 4 weeks and discharged on day 33 after the procedure with good progression. A 3-year follow-up CT scan showed aneurysm reduction and no recurrent infections. This case report highlights that TAE can be a treatment option for patients with an infected artery aneurysm.

## Introduction

Infected aneurysms account for 3.3% of all aneurysms [1], and infected aneurysms of the aorta and iliac arteries constitute 0.6–1.3% of all such aneurysms [2], [3], [4]. The in-hospital mortality rate due to ruptured aneurysms is 75%-100%, making it a life-threatening disease [[5], [6]]. Standard treatment for infected aneurysms is the removal of the infected lesion and surgical revascularization [2]. Endovascular treatment is proposed as a bridging procedure to a later open surgical repair in patients with hemodynamic instability [7]. While TAE is commonly performed for infected aneurysms in the cerebral artery because of its less invasive nature [8], its use in the trunk is limited. To the best of our knowledge, there are no reports of TAE for infected internal iliac artery aneurysms with good long-term outcomes [[9], [10], [11]]. Here, we present a case of an infected internal iliac artery aneurysm in a patient at high operative risk treated with TAE and appropriate antibiotics, resulting in no recurrent postoperative infection during long-term follow-up. Written informed consent was obtained from the patient.

## Case report

**Fig. 1:**
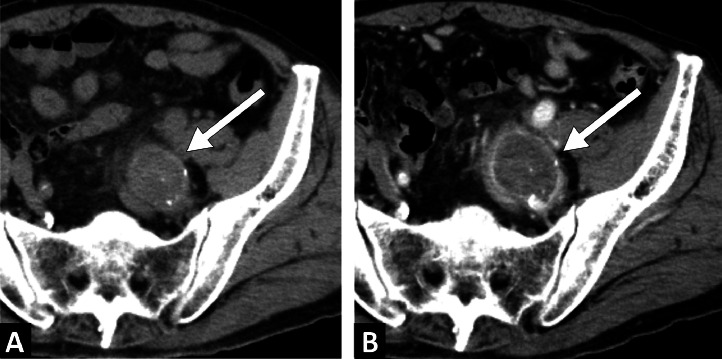
(A) Computed tomography initially conducted at another hospital showing a left internal iliac artery aneurysm with a maximum short diameter of 30 mm (arrow). (B) Computed tomography angiography conducted one week after the initial scan showing the left internal iliac artery aneurysm rapidly enlarged to a maximum short diameter of 38 mm (arrow).

**Fig. 2:**
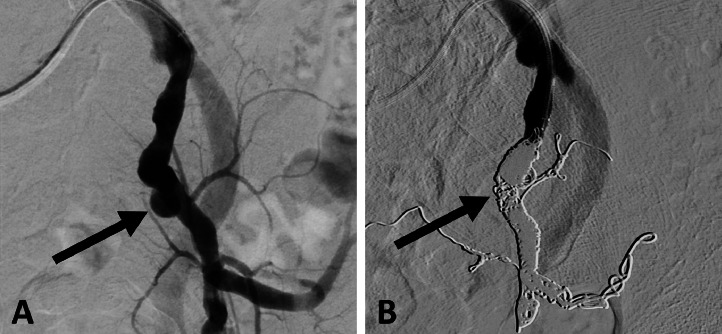
(A) Left internal iliac angiogram showing the aneurysm (arrow). (B) Digital subtraction angiography in the right anterior oblique 30-degree view showing no opacification of the aneurysm (arrow) after transarterial embolization.

**Fig. 3:**
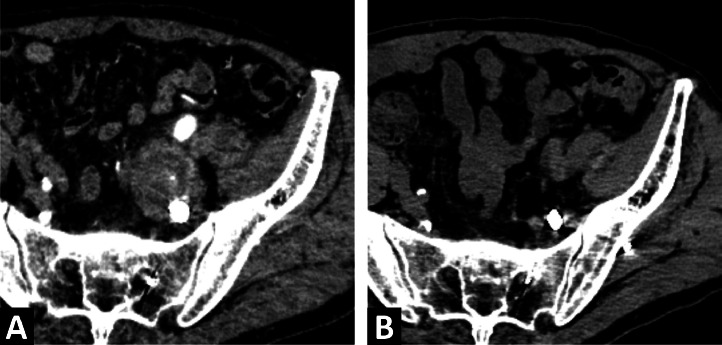
(A) Computed tomography angiography on day 6 after the procedure showing no enhancement of the aneurysm. (B) Three-year follow-up computed tomography angiography showing shrinkage of the aneurysm.

An 85-year-old man with a history of percutaneous coronary intervention for angina pectoris underwent computed tomography (CT) at a previous hospital to investigate the cause of fever and left lower abdominal pain, which revealed a 30-mm left internal iliac artery aneurysm (Fig. 1A). One week later, CT angiography revealed that the aneurysm diameter had increased to 38 mm (Fig. 1B). As the patient was transferred to our hospital for fever, abdominal pain, elevated white blood cell counts, and rapid expansion of the aneurysm, an infection of the aneurysm was suspected. On arrival, his vital signs were stable. Physical examination revealed left lower abdominal pain, and laboratory test results showed anemia and elevated levels of C-reactive protein. Surgical treatment was planned to manage the infected aneurysm; however, the patient's age and general condition made the surgery high-risk. Therefore, an emergency TAE was performed on the same day. A puncture was made using the right femoral artery approach. A 4-Fr Rösch inferior mesenteric catheter (Cordis, Miami, FL) was inserted into the left common iliac artery, and a 6-Fr destination guiding sheath (Terumo, Tokyo, Japan) was inserted into the left common iliac artery. A 5.2-Fr Selecon MP catheter (Terumo) was inserted into the superior and inferior iliac arteries and embolized using AZUR CX35 coils (Terumo) (one 8 × 24 cm, 3 6 × 17 cm). Coiling of the lateral sacral and iliolumbar arteries to the main trunk of the internal iliac artery with a total of 7 60-cm packing coils was achieved. The proximal left internal iliac artery was embolized using Target 360 soft detachable coils (Stryker Neurovascular, Fremont, CA) (1 7 × 20 cm, 1 8 × 30 cm). TAE was performed to isolate the left internal iliac artery aneurysm, and digital subtraction angiography showed no opacification of the aneurysm (Fig. 2). CT on day 6 after the procedure revealed no enhancement of the aneurysm (Fig. 3A). The patient was administered 3 g/day meropenem (Meropen, Sumitomo Pharma Co., Osaka, Japan) starting from the day of admission. Meropenem administration ended at 4 weeks with an improvement in the inflammatory response. No organisms were isolated from the blood culture. The patient was discharged on day 33 after the procedure with good progression. A 3-year follow-up CT scan showed aneurysm reduction and no recurrent infections (Fig. 3B).

## Discussion

The present case of emergency TAE for an infected internal iliac artery aneurysm in an older patient was successfully treated without recurrent infection. Dang et al. conducted a retrospective observational study on 26 patients with infected abdominal aortic aneurysms and reported that perioperative blood loss was remarkably higher and intensive care unit stay was significantly longer in the open repair group than in the stent graft group (11.8 ± 23.9 days vs. 0.46 ± 1.1 days, *P* < .01) [12]. There were no significant differences in the rates of retreatment, rehospitalization, or mortality between the 2 groups in terms of prognosis at 30 days and 1 year [12]. Similar to stent grafts, TAE is considered less invasive than open surgery. While there may be concerns regarding the use of TAE on infected lesions due to the possibility of exacerbating the infection with the introduction of a foreign object [11], the successful long-term outcome without recurrent infection in this case suggests that TAE and appropriate antibiotics could be a viable treatment option for patients with an infected aneurysm in whom surgical repair is difficult.

## Conclusion

We report a case of an infected internal iliac artery aneurysm in a patient at high operative risk treated with TAE who remained without recurrent postoperative infection after a long-term follow-up. TAE and appropriate antibiotics can be a potentially lifesaving treatment option for patients with an infected internal iliac artery aneurysm.

## Ethical approval

For this type of study, formal approval is not required.

## Patient consent

Written informed consent was obtained from the patient included in this study.
